# Non-nicotine constituents in cigarette smoke extract enhance nicotine addiction through monoamine oxidase A inhibition

**DOI:** 10.3389/fnins.2022.1058254

**Published:** 2022-11-24

**Authors:** Guanglin Liu, Ruiyan Wang, Huan Chen, Ping Wu, Yaning Fu, Kaixin Li, Mingda Liu, Zhihao Shi, Yuan Zhang, Yue Su, Lingxiao Song, Hongwei Hou, Qingyuan Hu

**Affiliations:** ^1^China National Tobacco Quality Supervision & Test Center, Zhengzhou, China; ^2^Key Laboratory of Tobacco Biological Effects, Zhengzhou, China; ^3^Joint Laboratory of Translational Neurobiology, Zhengzhou, China; ^4^National Institute on Drug Dependence and Beijing Key Laboratory of Drug Dependence, Beijing, China

**Keywords:** denicotinized cigarette, tobacco addiction, conditioned place preference, dopamine, non-nicotine constituents

## Abstract

Tobacco addiction has been largely attributed to nicotine, a component in tobacco leaves and smoke. However, extensive evidence suggests that some non-nicotine components of smoke should not be overlooked when considering tobacco dependence. Yet, their individual effect and synergistic effect on nicotine reinforcement remain poorly understood. The study herein focused on the role of non-nicotine constituents in promoting the effects of nicotine and their independent reinforcing effects. Denicotinized cigarettes were prepared by chemical extracting of cut tobacco, and the cigarette smoke extracts (CSE, used as a proxy for non-nicotine ingredients) were obtained by machine-smoking the cigarettes and DMSO extraction. The compositions of harmful components, nicotine, and other minor alkaloids in both cut tobacco and the CSE of experimental denicotinized cigarettes were examined by GC-MS, and compared with 3R4F reference cigarettes. individually and in synergy with nicotine were determined by conditioned place preference (CPP), dopamine (DA) level detection, the open field test (OFT), and the elevated plus maze (EPM). Finally, the potential enhancement mechanism of non-nicotinic constituents was investigated by nicotine metabolism and monoamine oxidase A (MAOA) activity inhibition in the striatum of mice and human recombinant MAOA. Thenicotine content in smoke from the experimental denicotinized cigarettes (under ISO machine-smoking conditions) was reduced by 95.1% and retained most minor alkaloids, relative to the 3R4F reference cigarettes. It was found that non-nicotine constituents increased acute locomotor activities. This was especially pronounced for DA levels in NAc and CPP scores, decreased the time in center zone. There were no differences in these metrics with DNC group when compared to the NS group. Non-nicotine constituents alone did not show reinforcing effects in CPP or striatum DA levels in mice. However, in the presence of nicotine, non-nicotine constituents further increased the reinforcing effects. Furthermore, non-nicotine constituents may enhance nicotine’s reinforcing effects by inhibiting striatum MAOA activity rather than affecting nicotine metabolism or total striatum DA content in mice. These findings expand our knowledge of the effect on smoking reinforcement of non-nicotine constituents found in tobacco products.

## Introduction

As the main psychoactive component of tobacco, nicotine is considered essential for dependence (Ashok et al., 2017). Most existing work on tobacco dependence has focused only on nicotine (Gellner et al., 2016), and nicotine is widely administered as a substitute of tobacco (Coe et al., 2005). However, nicotine alone cannot fully explain the strong dependence on tobacco in smokers. The effects on intracranial self-stimulation thresholds caused by nicotine are even weaker than very weak drugs like caffeine or phencyclidine (Bespalov et al., 1999), and nicotine requires more sessions than general addictive drugs to form a significant place preference (Piechota et al., 2010).

In an investigation of smokers (Rose et al., 2010), when comparing the attractiveness of non-nicotine constituents and nicotine, it was found that denicotinized cigarettes (used as a model of non-nicotine constituents) were self-administered more than other routes of nicotine administration (including intravenous injection of nicotine, nicotine patches, and sprays). This result demonstrates the smokers’ preference for non-nicotine constituents, but their abuse potential remains to be explored. In addition to nicotine, tobacco smoke contains more than 7,000 different compounds (Rodgman and Perfetti, 2008), some of which may affect neurochemistry or act as nicotine enhancers.

In terms of psychoactive effects, three major alkaloids in cigarette smoke (nornicotine, anabasine, and R-anatabine) have been reported to activate α4β2 nAChR (a major subtype of nicotinic acetylcholine receptor expressed in the brain) and could result in increased midbrain DA release (Hoffman and Evans, 2013; Wills and Kenny, 2021) similar to nicotine (McGranahan et al., 2011). Moreover, it has been reported that nornicotine, in concentrations much higher than found in tobacco smoke, can independently support weak intravenous self-administration in rats (Bardo et al., 1999).

Some other mechanisms may help explain the increased attraction of cigarettes. Phenylethylamine and benzaldehyde in cigarette smoke were reported to reduce the activity of CYP2A6 (Rahnasto et al., 2003), an enzyme responsible for about 80% of nicotine metabolism in humans: The inhibition of CYP2A5 [the ortholog of the human CYP2A6 gene in mice (Le Foll and Goldberg, 2005; Grabus et al., 2006)] significantly delayed nicotine metabolism and thus decreased nicotine intake behavior (Sellers et al., 2003; Damaj et al., 2007; Bagdas et al., 2014; Chen et al., 2020; Goyal et al., 2021). Studies have also reported that pretreatment with monoamine oxidase A (MAOA, the main enzyme of physiologically active monoamines) inhibitors could also promote nicotine self-administration (Guillem et al., 2005, 2006), and constituents like harman and norharman in cigarette smoke were reported to inhibit MAOA (Toorn et al., 2019).

For total cigarette smoke composition, numerous studies have shown that cigarette smoke extracts (CSE) were more addictive than nicotine alone at the same concentration (Brennan et al., 2014, 2015; Costello et al., 2014). CSEs have been widely used in toxicology research (DeMarini, 2004; Liu et al., 2007; Donate et al., 2021; Song et al., 2021), and these extracts provide another opportunity to study the collective contribution of many non-nicotine constituents in the aerosol condensate matrix. Although isolating CSEs can be difficult, their study can be more directly related to the key questions in clinical cases.

Conditioned place preference (CPP) is a classical method in drug reinforcement research (Le Foll and Goldberg, 2005; Grabus et al., 2006), and was used for assessing addiction-related behaviors, as well as for quantifying the rewarding effects of substances (Cunnigham et al., 1993; Cunningham et al., 2006). The DA neurotransmitter system is central to addiction, and psychostimulants produce an abnormal release of DA in the midbrain of animals and humans (Di Chiara, 2000; Dani, 2003; Hyman et al., 2006; Benowitz, 2010; Deadwyler, 2010). The effect of cigarettes (Barrett et al., 2004; Brody et al., 2004) and other addictive drugs (Di Chiara et al., 2004) on the midbrain DA system function correlates with self-reported drug liking measurements in humans, suggesting that increased midbrain DA levels caused by psychoactive drugs are important indicators for reinforcing effects analysis. Moreover, the recently reported advanced monitoring measurement, a genetically encoded DA fluorescent biosensor (Sun et al., 2018), was able to circumvent previous problems, including the long sampling time (5–10 min interval) of microdialysis (Chefer et al., 2009), low detection limit, inability to distinguish catecholamine neurotransmitters of fast scanning cyclic voltammetry (Venton and Cao, 2020), and the biosensor also responded well to DA in both rodents and neurons *in vitro* (Sun et al., 2020). The sensing module based on natural membrane-targeted DA receptor ensures sufficient chemical specificity and an adequate level of detection. A conformational change in the receptor due to extracellular dopamine binding, Green Fluorescence Protein (GFP) domain that located in the third intracellular loop (Sun et al., 2020; Salinas et al., 2022).

In the present study, we used the CSE of denicotinized cigarettes (made of cut tobacco after chemical extraction) as a model for non-nicotine constituents. The CPP test and DA recordings were used to examine the reinforcing effects of the CSE on experimental denicotinized cigarettes. Nicotine metabolism and MAOA inhibition were examined to identify the potential mechanism of the enhancing effect of non-nicotine constituents on nicotine. Our results provide valuable insights into tobacco reinforcement by more clearly defining the roles of non-nicotine constituents in cigarettes.

## Results

### Chemical analysis of cut tobacco and smoke of experimental cigarettes

**FIGURE 1:**
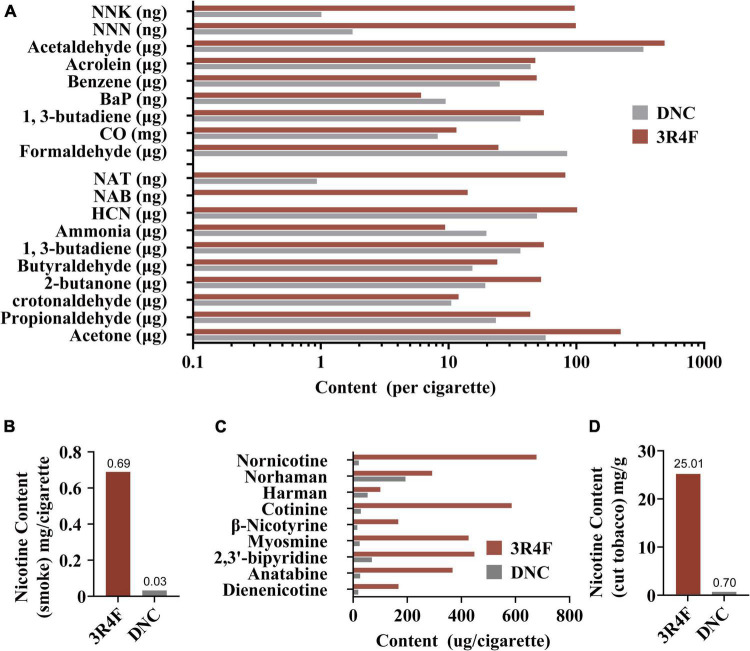
The contents of selected harmful substances in mainstream smoke extracts under ISO smoking, including 9 priority harmful substances listed by the World Health Organization: NNK, 4-(methylnitrosamino)-1-(3-pyridyl)-1-butanone; NNN, N′-nitrosonornicotine; Acetaldehyde; Acrolein; Benzene; BaP, Benzo[a]pyrene; 1,3-Butadiene; CO, Carbon monoxide; Formaldehyde **(A)**. Nicotine contents in mainstream smoke extracts from ISO smoking **(B)**. Minor alkaloid contents in mainstream smoke extracts from ISO smoking **(C)**. Nicotine contents in cut tobacco **(D)**.

An overview of the composition analysis of smoke and cut tobacco between the experimental cigarettes and the 3R4F reference cigarettes can be found in Figure 1. The contents of most harmful substances in the smoke of the experimental cigarette were close to that of the 3R4F reference cigarettes (Figure 1A). Compared with the CSE of 3R4F reference cigarettes, a significant decrease was found in the nicotine contents (0.69 mg/cigarette to 0.03 mg/cigarette, Figure 1B) and most minor alkaloids (Figure 1C) in the CSE of the experimental cigarettes. Similarly, the nicotine content of the experimental cigarettes (0.70 mg/g) was lower than the 3R4F reference cigarettes (25.01 mg/g) (Figure 1D) in the cut tobacco.

### Behavioral tests

**FIGURE 2:**
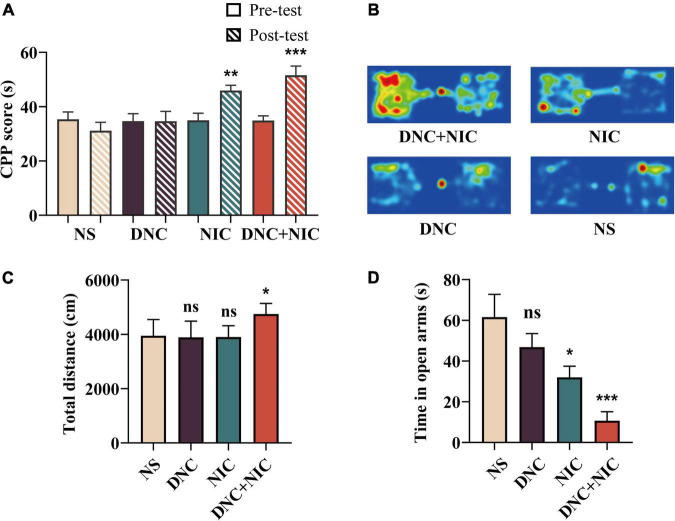
Non-nicotine constituents enhanced nicotine addiction. Conditioned place preference before and after training (*n* = 8), NIC effect *F*_1, 28_ = 26.180, *p* < 0.001, DNC effect *F*_1, 28_ = 2.171, *p* = 0.152; interaction *F*_1, 28_ = 0.124, *p* = 0.727 **(A)**. Heat map of the time spent in each area of the CPP chamber **(B)**. The total distance traveled in the open field (*n* = 8), NIC effect *F*_1, 20_ = 4.812, *p* = 0.040, DNC effect *F*_1, 20_ = 4.735, *p* = 0.042, interaction *F*_1, 20_ = 12.515, *p* = 0.002 **(C)**. Time in the open arms (*n* = 8), NIC effect *F*_1, 28_ = 19.696, *p* < 0.001, DNC effect *F*_1, 28_ = 5.931, *p* = 0.021, interaction *F*_1, 28_ = 0.196, *p* = 0.661 **(D)**. One-way analysis of variance (ANOVA) *post hoc* test for **(A,C,D)**: *p < 0.05, **p < 0.01, ***p < 0.001.

Compared with the NS group (animals administrated normal saline), the DNC + NIC (***p < 0.001) and NIC (*p < 0.05) groups showed significant CPP, and compared with the DNC group, the DNC+NIC (* *p* < 0 01) group and the NIC (* *p* < 0 05) group showed obvious CPP (Figures 2A,B), while there was no significant difference (ns, *p* > 0.05) when comparing the DNC group (animals administrated denicotinized cigarette CSE) with the NS group. The open field test, conducted immediately after injection, indicated that only the DNC + NIC treatment showed an increased effect (**p < 0.01), compared with the DNC + NIC group, the total activity distance of the DNC group and the NIC group was less, and the difference was statistically significant (*p < 0.05). while the other treatments had no significant (ns, *p* > 0.05) effect on locomotor activities (Figure 2C). The elevated plus maze test (8 h after OFT) showed a state of intense anxiety in the group of the DNC + NIC (***p < 0.001) and the NIC (*p < 0.05), compared with the DNC group, the DNC + NIC group stayed in the open arm for a shorter time, indicating that the DNC+NIC group was more anxious, and the difference was statistically significant (Figure 2D).

### Dopamine level detection

**FIGURE 3:**
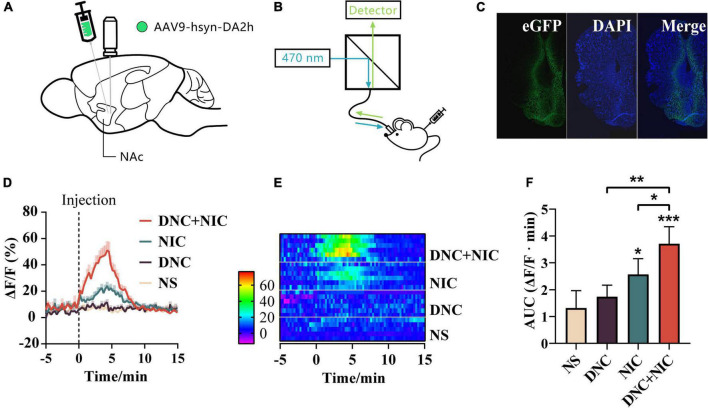
Real-time DA level during drug exposure. Schematic illustration of virus injection and the optical fiber embedment **(A)**. Schematic diagram of an optical fiber recording device **(B)**. The expression of GRAB_DA2h_ was tracked on the coronal section in the nucleus accumbens **(C)**. DA signals in NAc of drug treatments (baseline: −5 to 0 min. 0 min: injection time point, s.c.) (*n* = 6). Mean ± SEM overlaid for each group **(D)**. Heat maps of DA level recording related to drug administration **(E)**. The area under the curve (AUC) of the DA signal, Mean ± SEM, NIC effect *F*_1, 20_ = 50.145, *p* < 0.001, DNC effect *F*_1, 20_ = 12.470, *p* = 0.002, interaction *F*_1, 20_ = 1.960, *p* = 0.177, one-way analysis of variance (ANOVA) *post hoc* test for *p < 0.05, **p < 0.01, ***p < 0.001 **(F)**.

Two weeks after unilateral microinjection of adeno-associated virus (AAV) into the NAc (Figure 3A), and the green fluorescence intensity was measured using by the multichannel fiber photometry system, which reflected the DA level (Figure 3B). Mice showed strong immunohistochemical localization of the DA fluorescence biosensor GRABDA2h in NAc slices (Figure 3C).

The real-time DA levels were tested during injections (9^th^ day, 8 h after post-test). The results showed that compared with NS, NIC enhanced the DA level [two-way ANOVA; *F* (1, 5) = 6.96, *p < 0.05; Figure 3D], while DNC showed no enhancement of the DA level [two-way ANOVA; *F* (1, 5) = 0.24, ns, *p* > 0.05; Figure 3D]. DNC + NIC enhanced the DA level [two-way ANOVA; *F* (1, 5) = 30.20, **p < 0.01; Figure 3D], and the addition of DNC significantly enhanced the DA level induced by NIC [two-way ANOVA; *F* (1, 5) = 39.15 **p < 0.01; Figure 3D] as was also the case with CPP scores.

The DNC + NIC group induced stronger DA release than the NIC group (Figure 3D). These results are also presented by the heat-map graph (Figure 3E). The AUC (area under curve) induced by the DA in DNC (** *p* < 0.01) group and NIC (* *p* < 0.05) group was significantly lower than that in the DNC + NIC group. The DA-released AUC (area under the curve) induced by the DNC group (mean = 173.7) was higher than that of the NS group (mean = 131.4), but there was no statistical difference (*p* > 0.05, Figure 3F). The results indicate that non-nicotine constituents may potentiate nicotine reinforcing effects by a mechanism of increasing the nicotine-induced DA release.

### Investigation of potential mechanisms

#### The influence of non-nicotine constituents on nicotine metabolism

**FIGURE 4:**
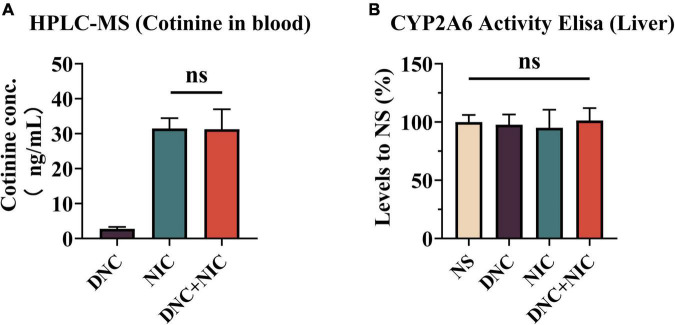
Non-nicotine constituents did not affect nicotine metabolism. Plasma cotinine (ng/mL) at 30 min after subcutaneous administration [*n* = 6, **(A)**]. The activity of CYP2A5 in mouse liver and compared to the NS group, NIC effect *F*_1, 12_ = 0.003, *p* = 0.957, DNC effect *F*_1, 12_ = 0.033, *p* = 0.859, interaction *F*_1, 12_ = 0.161, *p* = 0.695 with one-way analysis of variance (ANOVA) *post hoc* test [*n* = 6, **(B)**].

To examine the influence of non-nicotine constituents on nicotine metabolism, the blood concentrations of cotinine (the main nicotine metabolite) were determined after the injection of the drug. The results indicate that the DNC + NIC group was not significantly different from NIC (*p* = 0.9995, one-way ANOVA, Figure 4A). Compared with the DNC group, the level of cotinine in the DNC + NIC (**p < 0.01) group and the NIC (**p < 0.01) group was higher, and there was statistical significance. The activity of CYP2A5 (the principal metabolism enzyme of nicotine in mice) in the liver showed that levels were not significantly different when NS was compared with DNC + NIC, NIC or DNC (DNC vs. NS, ns, *p* > 0.05;NIC vs. NS, ns, *p* > 0.05; DNC + NIC vs. NS, ns, *p* > 0.05 Figure 4B). Additionally, there was no significant difference among the other groups (DNC vs. DNC + NIC, ns, *p* > 0.05; DNC vs. NIC, ns, *p* > 0.05; Nic vs. DNC + NIC, ns, *p* > 0.05 Figure 4B).

#### Total dopamine amount and dopamine metabolism in striatum

**FIGURE 5:**
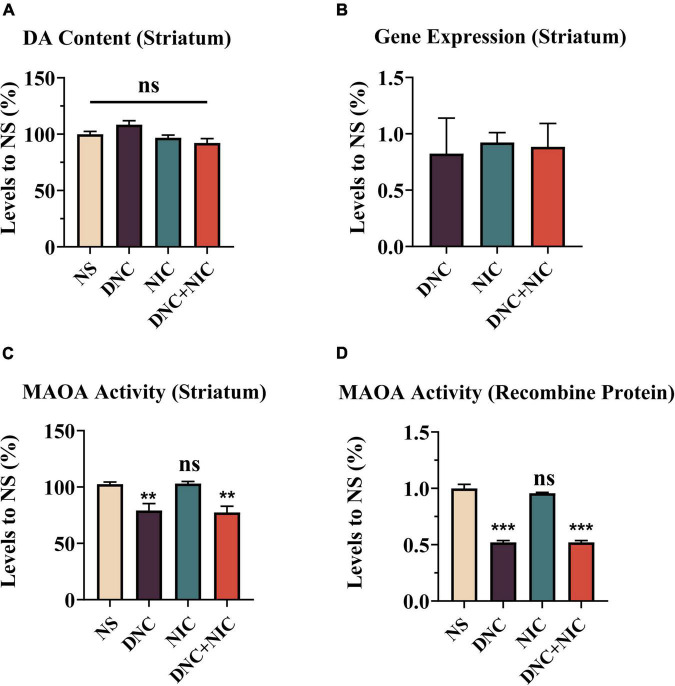
Administration of drugs did not change total striatum dopamine or MAO gene expression. DA concentration in striatum homogenate, NIC effect *F*_1, 32_ = 9.764, *p* = 0.004, DNC effect *F*_1, 32_ = 0.379, *p* = 0.542, interaction *F*_1, 32_ = 4.394, *p* = 0.044 [*n* = 8, **(A)**]. MAOA and MAOB mRNA expression as determined by qPCR [fold-change mRNA expression normalized to the NS group, *n* = 3, **(B)**]. Effects of drugs on the activity of MAOA in mice striatum in comparison to the NS group, NIC effect *F*_1, 24_ = 0.020, *p* = 0.889, DNC effect *F*_1, 24_ = 31.835, *p* < 0.001, interaction *F*_1, 24_ = 0.066, *p* = 0.799 [*n* = 6, **(C)**]. Effects of drugs on the activity of recombinant MAOA (Human recombinant protease) compared to the NS group, NIC effect *F*_1,8_ = 0.935, *p* = 0.362, DNC effect *F*_1, 8_ = 434.542, *p* < 0.001, interaction *F*_1, 8_ = 0.935, *P* = 0.362 [*n* = 3, **(D)**]. One-way analysis of variance (ANOVA) *post hoc* test for **(A,C,D)**: **p < 0.01, ***p < 0.001.

Dopamine (DA) amount and DA metabolism may also affect DA levels, thus affecting tobacco reinforcement. No statistically significant difference in DA amount between these groups was evident (ns, *p* > 0.05, Figure 5A). Additionally, inhibition of MAOA was examined. Besides inhibiting MAOA activity, MAOA inhibition was also associated with its’ gene expression levels. Instead of causing changes in MAOA expression levels in striatum (Figure 5B), DNC (**p = 0.0024) and DNC + NIC (**p = 0.0012) inhibited MAOA activity (Figure 5C). NIC alone did not cause changes in MAOA activity (ns, *p* > 0.05), nor did it synergize with DNC (ns, *p* > 0.05 compared DNC group to the DNC + NIC group, Figure 5C). However, the activity of MAOA in NIC group was significantly higher than that in DNC group (**P < 0.01). The activity of MAOA in DNC + NIC group was significantly lower than that in NIC group (**P < 0.01). Similar to the results of MAOA in striatum, DNC and DNC + NIC were also inhibited human recombinant MAOA activity *in vitro* (DNC + NIC vs. NS, ***p < 0.001; DNC vs. NS, ***p < 0.001. Figure 5D), and NIC had no effect alone and did not synergize with DNC (NIC vs. NS, ns, *p* > 0.05; DNC vs. DNC + NIC, ns, *p* > 0.05 Figure 5D). Compared with NIC group, the MAOA activity of DNC group and DNC+NIC group was inhibited *in vitro* (* *p* < 0.001).

## Discussion

The present study provides important information regarding the potential to augment nicotine reinforcement of non-nicotine constituents in cigarette smoke by preparing denicotinized cigarettes and applying them to animal experiments.

In this study, the contents of 19-associated smoke harmful chemical compositions, nicotine, and other minor alkaloids of 3R4F reference cigarettes and denicotinized cigarettes were reported (Figure 1). The contents of some harmful substances in the CSE of denicotinized cigarettes were consistent with those found in 3R4F reference cigarettes (Figure 1A), except for lower concentrations of tobacco-specific nitrosamines [TSNA, including 4-(methylnitrosamino)-1-(3-pyridyl)-1-butanone, NNK; N′-nitrosonornicotine, NNN; N′-nitrosoanatabine, NAT and N-nitrosoanabasine, NAB]. This is the characteristic difference between Chinese Virginia cigarettes (mainly containing flue-cured tobacco) and 3R4F reference cigarettes (blended cigarettes) (Xiong et al., 2010; Zhang et al., 2018) which is also the reason for the difference in minor alkaloids. To better understand the non-nicotine constituents in this study, the nicotine content of the cut tobacco in the experimental cigarettes was reduced to 0.7 mg/g, which was 2.8% of the 3R4F standard cigarette level (Figure 1B), and was close to 0.4–0.6 mg/g (Bespalov et al., 1999; Rodgman and Perfetti, 2008; Piechota et al., 2010; Rose et al., 2010; Ashok et al., 2017) in other studies of denicotinized cigarettes. Thus, the observed differences in smoke chemistry between 3R4F and denicotinized cigarettes can be attributed to differences in cigarette design and processes used to reduce nicotine content.

Despite significant inhibition of MAOA activity (Figures 5C,D), individual non-nicotine constituents caused no DA increase (Figures 3D–F). This finding was consistent with the work reported by Marti et al. (2011) on HEK293-α4β2 cells, which showed CSE did not cause additional α4β2-nAChR activation compared to nicotine, despite containing non-nicotine constituents. Our results differed from other studies when investigating the induced DA release effect of several minor alkaloids in smoke components in the NAc (Dwoskin et al., 1995; Arib et al., 2010) due in part to their much higher dose of administration.

The non-nicotine constituents showed no reinforcing properties in mice (Figure 2), which was further demonstrated by the lack of locomotion (Figure 2C) and the lack of anxiety symptoms after withdrawal for 3 days (Figure 2D). This was consistent with other studies that showed that individual psychoactive alkaloids (Hoffman and Evans, 2013) or the cocktail (Clemens et al., 2009) (anabasine, nornicotine, cotinine, and myosmine) were unable to induce reinforcing effects based on CPP on the dose related to cigarette smoke context.

For the CSE of denicotinized cigarettes and nicotine, the effect on mice seems to be in conflict with the increased attraction compared to nicotine administration in the study by Rose et al. (2010). This difference might be attributed to the prior smoking experience of the human subjects and the unique sensory attributes (Rose, 2006), including the “throat hit,” the sight, taste, and smell of cigarettes, the contexts cues (White et al., 2020), and the social and cultural factors (Stewart et al., 2015; Boudreau et al., 2016), which are considered essential factors of tobacco use (Wang et al., 2018). These factors further demonstrate the complexity of tobacco reinforcement and the importance of studying pharmacological and non-pharmacological non-nicotine factors.

The CPP test manifested the reinforcing effects of the rewarding stimulus (Tzschentke, 1998; Paredes, 2009), and the DNC + NIC group (mean = 0.52) showed longer time spent in the drug-paired environment than the NIC group (mean = 0.46) (Figures 2A,B), which implied the possible promotion of DNC in the presence of nicotine. A similar trend was found in DA release (Figures 3D–F). When administrated with nicotine, the peak value of abnormal release of DA in the NAc was about 135% above the base level, which was similar to other studies using microdialysis (Rada et al., 2001; Ding et al., 2022); this observation supported the reliability of using the DA biosensor to monitor drug-induced DA release, while DNC further enhanced the DA peak value of nicotine to 157% (Figure 3D).

The reinforcing effect and DA level induced by DNC + NIC were greater than the sum of the two parts, suggesting that the relationship of the DNC group to nicotine was synergistic rather than additive, and the synergistic effect was further supported by the unique enhancement of the DNC + NIC group in locomotor activity compared to the NIC group (Figure 2C) and anxiety performance (Figure 2D). The results show that there was no difference in blood cotinine concentration between the DNC + NIC and NIC groups (Figure 4A), and the same activity of CYP2A5 between the DNC and NS groups excluded pharmacokinetic factors. However, as compared to the addition of CSE into NS, the MAOA inhibition of non-nicotine constituents might be an important factor for the enhancement on nicotine, which would lead to stronger DA release and reinforcement properties. This is consistent with previous studies using the MAOA inhibitor (Guillem et al., 2005; Lotfipour et al., 2011; Smith et al., 2015, 2016) or CSE of normal nicotine content cigarettes (Costello et al., 2014; Hall et al., 2014; Gellner et al., 2016; Cross et al., 2020), but it is worth highlighting that this study complemented the effects of the mixture of non-nicotine constituents.

Similarly, nicotine transdermal patch supplementation reduced withdrawal symptoms in subjects who switched to denicotinized cigarettes (Buchhalter et al., 2005; Donny and Jones, 2009). It must be acknowledged that chemical extraction did not completely remove nicotine, but the CSE of denicotinized cigarettes with residual little nicotine in this study did not cause the above enhancement, which indicate there may be a concentration threshold for most behavioral or physiological actions. Although 0.5 mg/kg dose nicotine is widely used and caused the strongest CPP response (Grabus et al., 2006; Walters et al., 2006), the limitations of evaluating the effects of single nicotine dose in this study warrant future work. Furthermore, we also investigated the influence of repeated exposure to different formulated injections on DA levels, and the result showed no difference in striatum DA measurements between the different groups (Figure 5A), which was similar to results after chronic exposure (Pietilä and Ahtee, 2000).

Genetically encoded DA sensors (Sun et al., 2018) were successfully used to monitor the DA levels and exhibited excellent performance in terms of long-range stability and reliability. The results suggest that the DA-releasing ability of drugs was synchronized with the CPP reinforcing effects test (Figures 2, 3), which demonstrated the great potential of DA detection as an indicator for evaluating reinforcing properties. Moreover, based on its much higher sampling frequency, the biosensor can more realistically reflect the release dynamics of DA (Salinas et al., 2022), especially when exploring the drug-related DA dynamics even at sub-second timescales.

The results of this study suggests that denicotinized cigarettes have the potential to be the model applied in the study of non-nicotine constituents, but we also note the limitations of residual nicotine in these cigarettes. Additionally, the DNC group in the present study represents only initial user, and the study of subjects with a smoking history of normal nicotine content cigarettes deserves further investigation.

## Conclusion

This study utilized denicotinized cigarettes as a model of non-nicotinic constituents and assessed their abuse reinforcing effects and effects in mice. The molecular and behavioral results obtained in the present animal study suggest that non-nicotinic constituents alone could inhibit MAOA activity but could not induce CPP or promote extra DA release. Nicotine could induce CPP or extra DA release, but it was far less effective than CSE from normal nicotine content cigarettes. In contrast to the widely known potentiation properties of non-nicotine ingredient mixtures, this study is the first to show their diminished effects when they are applied in the absence of nicotine. These results indicate that exploring the interaction between nicotine and non-nicotine components may help researchers better understand why tobacco in the form of cigarettes is highly addictive.

## Materials and methods

### Study design

**FIGURE 6:**
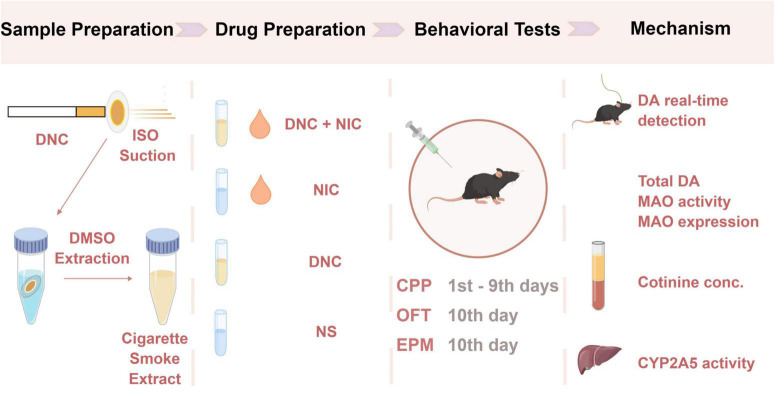
A brief outline of the study design.

The study design was performed as shown in Figure 6.

Cigarette sample preparation: Denicotinized cigarette smoke extracts were obtained using cigarette suction and DMSO extraction. Components of tobacco and smoke were analyzed by GC-MS (gas chromatography-mass spectroscopy). Detailed steps are described above.

Cigarette smoke extracts (CSE) stocks preparation and grouping: Given the different contents of individual constituents among the experimental cigarettes and 3R4F cigarettes, nicotine was added to the CSE of denicotinized cigarettes (named DNC) to a final concentration that was consistent with the nicotine content of 3R4F cigarette CSE (named DNC + NIC) and represented the normal nicotine content of cigarettes. Likewise, NIC was obtained by adding the same amount of nicotine into DMSO, and NS means DMSO control. CSE stocks were dissolved in physiological saline before injection.

Behavioral tests: Four groups (*n* = 32, male mice) received different injections and were subjected to behavioral tests to determine the rewarding and reinforcing effects of the injection components. CPP (1^nd^–9^th^ days) was used to assess addiction-related behaviors and partially quantify the rewarding effects of substances. OFT (10^th^ day) was used to test locomotion in mice. EPM (10^th^ day) was used to monitor anxiety-like behavior in mice.

Potential mechanism research: Four groups of mice (*n* = 24, male) received different injections, and a genetically encoded DA sensor was used to monitor the DA dynamics on the 7^th^ injections. Blood was collected and analyzed by LC-MS to measure the blood cotinine concentration.

Thirty minutes after the administration (8^th^ day), 24 animals were sacrificed by intraperitoneal injection of 2,2,2-tribromoethanol (Aibei Biotechnology), and blood, liver, and brain samples were collected.

The activity of CYP2A5, the total amount of DA, and the activity of MAOA in the brain of mice treated with different drugs were measured.

### Cigarette preparation and smoking

Based on increasing polarity, petroleum ether, chloroform, acetone, and water were used to extract the cut tobacco (Yunnan, China) components by extraction pot (RTN-6.0, Henan, China). The acetone extract, the chloroform extract, and the petroleum ether extract were backfilled into the cut tobacco and packed in conventional tobacco tubes (84 × 24.0 mm), which were used in follow-up studies.

After incubation at 22 ± 1°C and 60 ± 3% relative humidity for 48 h, the smoke from DNC cigarettes and 3R4F reference cigarettes (University of Kentucky Lexington, KY, USA) was generated using a smoking machine (Model SML2000, Anhui, China) in accordance with the ISO 3308 Standard Smoking procedure: puff volume 35 mL, puff duration 2 s, 60 s time interval between puffs, and no ventilation holes were blocked (International Standard Organization, 2012). The mainstream cigarette smoke was collected on a 44 mm filter pad (Borgwaldt, Germany) and extracted with DMSO for DNC. For DNC + NIC, nicotine was added to a final concentration of 1.176 mg/mL (the same concentration as 3R4F CSE), and 1.176 mg/mL nicotine in DMSO for NIC (DMSO only for NS). All CSE were stored at −80°C.

### Gas chromatography-mass spectroscopy analysis of nicotine and minor alkaloids

The filter disc was extracted with a 5% NaOH aqueous solution for 30 min, then triethylamine dichloromethane and an internal standard were added and mixed. After centrifugation, the dichloromethane phase was collected with an organic phase filter and placed in a chromatographic analysis bottle for injection and analysis. One microliter was analyzed by GC–MS. Separation was obtained with a DB-5MS capillary column (30 cm, 0.25 mm id, and 0.25 μm df). The splitless mode was used, with an inlet temperature of 280°C and an oven temperature program increasing from 100 to 155°C and a final temperature of 280°C and a total run time of 10 min.

### Drugs

Each day’s CSE solution was prepared fresh before experimentation to minimize differences. Briefly, the stock solutions of NIC and DNC + NIC were diluted in physiological saline at the required concentration (0.5 mg/kg) for subcutaneous injection (s.c.) and adjusted to pH 7.2–7.4. DNC and NS were made in the same way. Human Monoamine A Oxidase (recombinant, expressed in baculovirus-infected BTI insect cells) was purchased from SIGMA (M7316).

### Subjects tables

Male C57BL/6 mice, 6 to 8 weeks of age, were obtained from Charles River Animal Technology (Beijing, China). Mice had free access to food and water with a 12h/12h light/dark cycle throughout the experiment. After the mice were acclimated to the colony for at least 7 days, the test was initiated during the light phase. The experimental procedure was carried out as described by the National Institutes of Health Guidelines for the Care and Use of Laboratory Animals, and was approved by the Experimental Animal Management and Ethics Committee of the China Tobacco Quality Supervision and Inspection Center.

### Conditioned place preference

Eight 3-compartment chambers (Mobile Datum, Shanghai) were used to measure the locomotor activity and CPP. Sliding guillotine doors were used to separate the three compartments. The middle compartment (70 × 180 × 200 mm) had a blue PVC floor and walls. The end compartments (170 × 180 × 200 mm) provided different visual and tactile cues; one compartment (chambers without drugs) had black walls with a PVC strip hole floor and the other one (chambers with drugs) had white walls with a circular hole floor.

The nicotine injection dose of the DNC + NIC and NIC groups was 0.5mg/kg (s.c.) (Klemperer et al., 2019). To determine baseline preferences, the animals were placed in the middle chambers (acclimation period, 1min) and allowed to explore freely in the three chambers on the 1*^st^* pre-test day. From 2^nd^ to 8^th^ days, mice were placed in one side of the chamber for 15 min after injection. Animals received saline injections in the black chambers in the morning and drug injections in the white chambers in the afternoon, with the order of injections switched every day. Post-test were performed on the on the 9^th^ day, and the percentage of time spent in the drug-paired boxes was defined as the CPP scores.

### Open field test

The open field test was used to evaluate the motor ability of the different mouse experimental groups on the day after the post-test (10^th^ day). The test device was a gray frosted opaque acrylic glass box with an upper opening of 40, 40, and 40 cm. The illumination source was a 20W red fluorescent lamp placed 100 cm above the floor. The open field experiment was performed as previously described by JOVE (Journal of Visualized Experiments) (Cassidy et al., 2018). After drug injection, the animals were placed in the center of the open field and allowed to move freely for 10 min.

### Elevated plus maze

After 8 h of OFT, when the mice were experiencing withdrawal from nicotine, mice were subjected to the EPM test. The experimental device was a 5 × 5 cm central axis structure, with four 50 cm-high arms and every two adjacent arms were vertical. Two closed arms were surrounded by a high wall of 30 × 5 × 15 cm, while the other arms were open (30 × 5 × 1 cm). The light source was a 20W red fluorescent lamp located 100 cm above the maze. The mice were placed in the center of the maze with their head facing one open arm and were allowed to explore freely for 10 min. The time spent on opening and closing the arm was taken as a standard anxiety index. Smart 3.0 (Panlab, Harvard Apparatus, USA) was used for behavioral analysis of OFT and EPM experiments. The apparatus was cleaned thoroughly between trials with a 75% alcohol cotton cloth to eliminate any odor effect.

### Surgery

To perform DA recording during drug administration, AAV with GRABDA2h (Dopamine receptor with green fluorescent protein) was injected into the NAc, followed by optical fiber implantation to deliver excitation and emission light that reflect DA levels. Mice were anesthetized by intraperitoneal injection with a 0.2 mL/10 g tribromoethanol solution, fixed on a brain stereotactic instrument equipped with a circulating water insulation system, and erythromycin ointment was applied to the eyes of mice to avoid injury from surgical glare. The incision region was shaved and iodophor was applied for preoperative disinfection. After incision, mice were adjusted to align bregma (the intersection of the coronal suture and sagittal suture) and lambda points (the intersection of herringbone suture and sagittal suture) until the error did not exceed 0.03 mm. According to the NAc brain area [anterior and posterior (AP): 1.4 mm, medial and lateral (ML): ± 1.0 mm, dorsal and ventral (DV): −3.9 mm], a small hole was drilled in the skull. A fine glass electrode tube connected to a micro syringe pump was slowly lowered into the target brain area through the hole, and 250 nL of AAV9-hsyn-DA2h (Vigene Biosciences, Shandong, China) was injected at a speed of 25 nL/min. After the injection, the glass tube remained at the injection site for another 5 min, and was then slowly removed. An optical fiber was inserted 50 μm above the injection site, and a light curing resin was applied and cured with a UV lamp for 10–15 s to fix the optical fiber, with dental cement applied for further fixation. The animals were removed and placed in an incubator for animal surgery recovery. After the operation, the animals were raised separately and were allowed to recover for 2 weeks.

### Immunohistochemistry

After anesthesia, animals were intracardially perfused with 50 mL 1 × PBS and 50 mL 4% PFA/1 × PBS solution for histological analysis. After perfusion, the brain was collected, fixed in 4% paraformaldehyde at 4°C for 4 h, and gradually dehydrated in 10%, 20%, and 30% (w/v) sucrose in PBS. Then, the brains were immediately frozen in an OCT embedding medium and were cut to 20 μm slices. The thicknesses were incubated with Rabbit anti-GFP antibody (1:500) at 4°C overnight and then incubated with Alexa488 (Thermo) coupled with Goat anti-rabbit secondary antibody (1:500) for 1.5 h. DAPI was incubated for 5 min and cleaned for nucleus observations. Images were obtained on a Zeiss microscope and analyzed on ImageJ software.

### Dopamine level recording

Mice were acclimatized for 2 weeks prior to experiments for AAV expression. The formula △F/F(F−F_0_)/F_0_ was used to calculate differences in fluorescence, where F represents the current fluorescence intensity and F_0_ represents the baseline fluorescence. Before the experiment, mice were placed in the recording environment for 30 min to adapt, and the environment was kept quiet and dark to minimize any interference.

### Analysis of blood cotinine

Blood samples were collected from the mouse eye orbit. Samples were placed in a 1.5 mL centrifuge tube containing 20 μL heparin sodium, centrifuged immediately, and the supernatant was aspirated. The cotinine internal standard working solution was added to the supernatant followed by methanol, then vortex shaken, centrifuged, and placed in a chromatographic analysis bottle before injection and analysis.

HPLC and mass spectrometry were carried out following previous methods, with modifications (Faulkner et al., 2017). High Performance Liquid Chromatography (HPLC) and mass spectrometry were carried out following previous methods, with modifications (Faulkner et al., 2017). Briefly, complete column separation was performed on an ACQUITY UPLC-BEH-HILIC HPLC column (Waters, 2.1 × 150 mm, 1.7 μm), and the column temperature was 40°C. The mobile phase consisted of 10 mmol/L ammonium formate (pH = 3.5) (A) and pure acetonitrile (B) at a flow rate of 0.7 mL/min.

### Monoamine oxidase A activity, qRT-pCR and dopamine content ELISA kit

The striatum of mice was excised with a mold in a buffer solution at 4°C, quickly frozen in liquid nitrogen, and stored at −80°C. A steel ball oscillating grinder (oscillation for 5 s static for 5 s, repeat five times) was used to obtain brain tissue homogenate. Brain tissue was lysed with RIPA buffer (1 mL/100 mg of brain tissue), then transferred to a centrifuge tube and mixed thoroughly by pipetting up and down. After 5 min at room temperature, the homogenate was centrifuged (11,000 rpm, 10 min, 4°C). The supernatant was carefully removed and transferred to a new centrifuge tube. Recombinant human MAOA was dissolved in 1 × PBS.

The DA content and MAOA activity levels in mice striatum were measured using an enzyme linked immunosorbent assay (ELISA) kit (Shanghai Jiang Lai Biotechnology Co., Ltd., China) based on the manufacturer’s procedures. Quantitative Real-time Polymerase Chain Reaction (qRT-PCR) was performed using qRT-PCR kit (Accurate Biology) following the manufacturer’s instruction.

### Statistical analysis

All statistical data were analyzed by GraphPad Prism (Version 8.4, GraphPad Software Inc., USA) and were tested for normality. One-way analysis of variance (ANOVA) followed by Turkey’s test was used for all the analyses except DA curve comparisons (two-way ANOVA). The fluorescence intensity was determined according to the equation, △F/F(F−F_0_)/F_0_ with SEM. Data are presented as the mean ± SEM, and were analyzed with two-way analysis of variance (ANOVA) followed by Bonferroni’s *post hoc* test and one-way ANOVA followed by Turkey’s test. *p* < 0.05 indicates a significant difference, and different significance levels are marked as *p < 0.05, **p < 0.01, and ***p < 0.001.

## Data availability statement

The original contributions presented in this study are included in the article/supplementary material, further inquiries can be directed to the corresponding authors.

## Ethics statement

The animal study was reviewed and approved by Laboratory Animal Management and Ethics Committee of China National Tobacco Quality Supervision and Test Center.

## Author contributions

GL, RW, HC, PW, HH, and QH conceived and designed the experiments. GL and RW performed the main experiments. ML and KL participated in assisting the ELISA experiments. YF, ZS, YZ, YS, and LS performed the cigarette suction and chemical detection experiments. GL, HC, and PW performed the data analyses and drafted the manuscript. GL and HC revised the manuscript. All authors have read and approved the manuscript.
